# The Treatment of Prisoners

**Published:** 1898-05-07

**Authors:** 


### Editor's Letter-Box.

[Our Correspondents are reminded that prolixity is a great bar to publication, and that brevity of style and conciseness of statement greatly facilitate early insertion.]

### THE TREATMENT OF PRISONERS.

"M.A." writes: In your remarks on the treatment of prisoners you speak of prisons as temporary places of confinement where a little hunger will do the strong and healthy no great harm. But Dartmoor, which was specially referred to, is a place for the confinement of persons in penal servitude, many of whom are "lifers," and the minimum period of detention is three years. What do you think of the want of sufficient food for this period? But I believe it is not the strong and healthy who suffer most. It is the weak and delicate who cannot eat the kind of food with which they are supplied. They are kept alive by repeated visits to the prison infirmary. In the case of the last convict who was shot dead while attempting to escape from Dartmoor no violence of any kind was offered to the warders, and I believe the same remark is applicable to the last prisoner, who was flogged on recapture (not at Dartmoor).

*** We have referred this letter to the writer of the article, who responds as follows: I am obliged by your letting me see your correspondent's letter. He does not seem to be aware that the food of prisoners is regulated by the length of their sentences, those undergoing long terms having much improved fare compared to those detained only for a short time. The term "lifer" is very seldom a practical reality; if the life convict behaves well he is released after a certain number of years. I deny absolutely, at least as regards English and Scottish prisons, that weak and delicate prisoners unable to eat the food provided are left in that condition; they are at once placed on hospital diet, and kept on it until their time expires, though they may never enter the infirmary at all. The fact, known in every police-court in the kingdom, that both men and women frequently commit some small offence—a petty theft or window breaking—in order to get into prison, which they prefer to the workhouse, does not look as if prisons were known to be such *infernos* as your correspondent seems to think. As to the penalties (well known to the convicts) for attempted escape, the danger to the warders is so great that it is necessary to make rules for their protection, even if on very rare occasions they escape injury. It seems to me, however, that your correspondent's views demand an examination of much wider questions, viz., Is there to be punishment for crime or not? Into this, however, I cannot now enter.

